# A Stable Genetic Transformation System and Implications of the Type IV Restriction System in the Nitrogen-Fixing Plant Endosymbiont *Frankia alni* ACN14a

**DOI:** 10.3389/fmicb.2019.02230

**Published:** 2019-09-24

**Authors:** Isaac Gifford, Summer Vance, Giang Nguyen, Alison M. Berry

**Affiliations:** Department of Plant Sciences, University of California, Davis, Davis, CA, United States

**Keywords:** *Frankia*, transformation, restriction enzymes, DNA Methylation, nitrogen fixation, root nodule, symbiosis, actinobacteria

## Abstract

Genus *Frankia* is comprised primarily of nitrogen-fixing actinobacteria that form root nodule symbioses with a group of hosts known as the actinorhizal plants. These plants are evolutionarily closely related to the legumes that are nodulated by the rhizobia. Both host groups utilize homologs of nodulation genes for root-nodule symbiosis, derived from common plant ancestors. The corresponding endosymbionts, *Frankia* and the rhizobia, however, are distantly related groups of bacteria, leading to questions about their symbiotic mechanisms and evolutionary history. To date, a stable system of electrotransformation has been lacking in *Frankia* despite numerous attempts by research groups worldwide. We have identified type IV methyl-directed restriction systems, highly-expressed in a range of actinobacteria, as a likely barrier to *Frankia* transformation. Here we report the successful electrotransformation of the model strain *F. alni* ACN14a with an unmethylated, broad host-range replicating plasmid, expressing chloramphenicol-resistance for selection and GFP as a marker of gene expression. This system circumvented the type IV restriction barrier and allowed the stable maintenance of the plasmid. During nitrogen limitation, *Frankia* differentiates into two cell types: the vegetative hyphae and nitrogen-fixing vesicles. When the expression of *egfp* under the control of the *nif* gene cluster promoter was localized using fluorescence imaging, the expression of nitrogen fixation in nitrogen-limited culture was localized in *Frankia* vesicles but not in hyphae. The ability to separate gene expression patterns between *Frankia* hyphae and vesicles will enable deeper comparisons of molecular signaling and metabolic exchange between *Frankia*-actinorhizal and rhizobia-legume symbioses to be made, and may broaden potential applications in agriculture. Further downstream applications are possible, including gene knock-outs and complementation, to open up a range of experiments in *Frankia* and its symbioses. Additionally, in the transcriptome of *F. alni* ACN14a, type IV restriction enzymes were highly expressed in nitrogen-replete culture but their expression strongly decreased during symbiosis. The down-regulation of type IV restriction enzymes in symbiosis suggests that horizontal gene transfer may occur more frequently inside the nodule, with possible new implications for the evolution of *Frankia.*

## Introduction

Bacteria in the genus *Frankia* form nitrogen-fixing root nodule symbioses with a group of host plants, the actinorhizal plants. The actinorhizal plants are evolutionarily closely related to the legumes within the Nitrogen-fixing Clade (NFC; Soltis et al., 1995). The bacterial symbionts are only distantly related to each, however, *Frankia* belongs to the phylum Actinobacteria, comprised of gram-positive bacteria with high-GC genomes. By contrast the rhizobia, symbionts of the legumes, are gram-negative proteobacteria with genomes generally closer to 50% GC. Additionally, *Frankia* is a multicellular, hyphal organism with a complex life cycle. In nitrogen limiting conditions, *Frankia* can produce stalked, ovoid to spherical structures that develop from branch hyphae called vesicles (Benson and Silvester, 1993). *Frankia* vesicles, known to be the sites of nitrogen fixation, are surrounded by a lamellar envelope comprised of hopanoid lipids (Berry et al., 1993) and have high rates of internal oxygen consumption through respiration. The vesicle envelope increases in number of layers in response to oxygen tension, thereby likely reducing the flow of oxygen into the vesicle interior to protect the nitrogenase complex from deactivation (Benson and Silvester, 1993). Thus, unlike the rhizobia-legume symbioses, where the host nodule tissue regulates the flow of oxygen during nitrogen fixation (Appleby, 1984), most *Frankia* are capable of fixing nitrogen in the vesicles in atmospheric oxygen conditions under nitrogen limitation (Benson and Silvester, 1993; Gtari et al., 2015). Finally, in symbiosis and *in vitro*, *Frankia* is capable of forming sporangia containing thick-walled spores (Benson and Silvester, 1993), which apparently serve as a resting stage and as a source of propagules that can enhance nodule numbers (Burleigh and Torrey, 1990).

Biological nitrogen fixation has potential applications in the development of minimal input strategies for more sustainable agriculture globally, and in nutrient-deficient soils (Gutierrez, 2012; Mus et al., 2016). Genetic improvement has the potential to introduce nitrogen-fixing symbioses into a wider range of crop plants once a broader understanding of the mechanisms involved in these symbioses is achieved (Mus et al., 2016). Actinorhizal and legume hosts share the Common Symbiotic Pathway, a signaling pathway at early stages of interaction that leads to the development of the nodule by the host after recognition of the symbiont (Oldroyd, 2013), in addition to several key gene orthologs involved in the process of nodule development (Battenberg et al., 2018; Griesmann et al., 2018). On the bacterial side the rhizobial signaling molecules that trigger nodulation in many of the legume hosts, called Nod factors, are synthesized by *nod* genes and have been extensively studied (Oldroyd, 2013). By contrast, the majority of *Frankia* genomes do not contain clear sets of *nod* gene homologs, with the exception of certain members of the cluster II *Frankia* whose genomes contain homologs of the rhizobial *nodABC* genes. These *nod* gene homologs are expressed in symbiosis (Persson et al., 2015). Other mechanisms have been found to trigger nodulation in some legumes by rhizobia, including an effector protein injected into the host by a type III secretion system (Okazaki et al., 2013), however genes responsible for these pathways lack homologs in *Frankia* genomes as well. Signals that elicit responses in host roots, including root-hair curling, an early step in some rhizobial and actinorhizal symbioses (Oldroyd, 2013), have been detected experimentally in some *Frankia* in clusters I and III. The structures of these molecules, however, have not been determined (Ceremonie et al., 1999; Cissoko et al., 2018).

The major advances in knowledge of rhizobial symbioses have been made possible by the development of genetic tools for dissecting the metabolic and regulatory pathways in the microsymbiont-host interactions. Long et al. (1982) originally demonstrated the role of the *nod* genes in symbiosis by complementing nodulation-deficient mutants of *Sinorhizobium meliloti* with *nod* genes encoded on a replicating, broad host-range plasmid. More recently, expression systems based on marker genes in rhizobia have enabled a wider-range of experiments including the tracking of microsymbionts during the host infection process (Gage, 2002; Fournier et al., 2015) and the identification of regulatory networks involved in symbiotic interactions (Spaepen et al., 2009). However, to date, there has been no stable electroporation-based genetic transformation system for *Frankia* (Kucho et al., 2009), and little understanding of what the barriers to stable transformation may be. Thus an electrotransformation system in *Frankia*, as reported here, has the potential to enable functional inquiries into the diverse mechanisms involved in establishing root-nodule symbiosis and maintaining biological nitrogen fixation in actinorhizal symbioses. These inquiries will, in turn, contribute to a wider understanding of the origins of root nodule symbioses and mechanisms of *Frankia* and rhizobial interaction with hosts in the NFC.

*Frankia* presents several barriers to transformation. Actinobacteria in general have low rates of homologous recombination due to competition between their homologous recombination pathway and a Non-Homologous End-Joining pathway (Zhang et al., 2012). Additionally, natural vectors for *Frankia* transformation are limited as no *Frankia* phages have been discovered (Simonet et al., 1990). Finally, its multicellular hyphae require experiments to be performed on hyphal segments rather than individual cells. Despite these barriers, it has been demonstrated that DNA can be electroporated into *Frankia* cells (Myers and Tisa, 2003), providing a potential avenue for genetic transformation. Furthermore, Kucho et al. (2009) reported initial success in integrating a non-replicating plasmid into the chromosome of *Frankia* sp. CcI3. However the recombined plasmid was lost in the following generation, limiting its further use in experiments.

Restriction enzymes pose a barrier to successful transformation in many bacteria due to their role in defense of digesting foreign DNA. In some actinobacteria, the use of unmethylated plasmids has increased transformation efficiency (Ankri et al., 1996; Molle et al., 1999), potentially due to the expression of type IV methyl-directed restriction enzymes. In the majority of bacterial taxa, DNA is methylated during replication by the methyltransferase Dam, to mark parent strands for DNA repair and excision of misincorporated bases from the daughter strand (Sanchez-Romero et al., 2015). The majority of actinobacteria, however, lack *dam* homologs (Sanchez-Romero et al., 2015) and an investigation of genomes of *Streptomyces*, *Rhodococcus*, and *Micromonospora* spp. found that these genomes were not methylated in the canonical Dam pattern (Novella et al., 1996). While actinobacteria have recently been shown to have a unique mismatch repair pathway that does not involve Dam (Castaneda-Garcia et al., 2017) it is not yet known how this pathway utilizes methylation, if at all.

In this study we report successful electrotransformation of *Frankia alni* ACN14a by using an unmethylated plasmid to circumvent the methylation-targeting restriction enzyme encoded in the ACN14a genome. This permitted the maintenance of plasmids derived from the very broad host-range replicating plasmid pIP501 (Kurenbach et al., 2003) in *F. alni* cultures for several months of continuous subculture, and implicates the type IV restriction enzyme as the major barrier to transformation. Use of a replicating plasmid also allowed the maintenance of the plasmid in *Frankia* without depending on its homologous recombination system. Bioinformatic analysis revealed a differential expression of type IV restriction genes between *F. alni* transcriptomes in symbiosis vs. culture, which has implications for acquisition of horizontally-transferred genes in endophytic environments by *Frankia*. Additionally, we show that e*gfp* expressed from a replicating plasmid can be used to label gene expression differences between *Frankia* cell types (vesicles vs. hyphae) during nitrogen fixation, opening up molecular genetics-based experiments, including marker gene expression, on *Frankia* symbioses in the future.

## Materials and Methods

### Restriction Enzyme Analysis

Restriction enzyme genes from all completely sequenced actinobacterial genomes available in the REBASE database (accessed 6/19/17) were downloaded with their annotations (Roberts et al., 2010). Restriction enzymes were categorized by enzyme types: I, II, III, or IV. Bacterial transcriptomes used in this study were downloaded from the NCBI GEO database (listed in Supplementary Table S1). Transcriptomes analyzed were chosen based on the following criteria: (1) transcriptomes were made from actively growing pure cultures, (2) the organism whose transcriptome was sequenced did not have any genetic manipulations, e.g., mutations or exogenous plasmids, and (3) media used for sequencing did not have any additional experimental compounds added including antibiotics or complex carbon sources. For comparisons between transcriptomes, expression levels for each gene were calculated as the percent of genes with lower expression than the gene of interest in each transcriptome (i.e., percentile ranking).

### Culture Conditions

*E. coli* strains DH5α and GM48 (*dam*- and *dcm*-) were grown in 50 ml Difco 1.5% (w/v) Luria Broth (LB) (Catalog #241420, Becton Dickinson, Franklin Lakes, NJ), pH 6.8, in 250 ml flasks at 37°C with shaking at 150 rpm overnight. Plates were made with 1.5% (w/v) Bacto Agar (Becton Dickinson, Franklin Lakes, NJ, United States, catalog #214010) in 1.5% LB, and incubated overnight at 37°C. All media were first sterilized by autoclaving for 30 min at 121°C.

*Frankia alni* ACN14a (Normand and Lalonde, 1982) was cultured in liquid BAPP media modified from Murry et al. (1984) by the addition of 5 mM pyruvate and 5 mM MOPS, adjusted to pH 6.7, in sterile plastic six-well plates (catalog #353046, Corning Inc., Corning, NY, United States) with 4 ml of media per well. We observed that *F. alni* ACN14a grew in the presence of tetracycline; thus tetracycline was added to a final concentration of 10 μg/ml to prevent contamination. (+)N media included 5 mM ammonium chloride as a nitrogen source whereas (–)N media had no added nitrogen source. For subculturing, hyphae were collected in sterile 2 ml microcentrifuge tubes with a 14G syringe needle, centrifuged at 10,000 rpm for 10 min in a tabletop microcentrifuge (model #5424, Eppendorf, Hamburg, Germany), and re-suspended in 1 ml fresh BAPP media. Cultures were then homogenized by passage through a 21G needle six times. Resulting homogenate equivalent to 50 μl packed-cell volume was added to 4 ml of fresh media in six-well plates and incubated at 28°C with shaking at 50 rpm. Cultures were routinely subcultured once per week. For selection of transformants, chloramphenicol was used at a final concentration of 25 μg/ml.

### DNA Extraction

Plasmids were purified from *E. coli* using a QIAprep^®^ Spin Miniprep Kit (Catalog #27106, Qiagen). Two milliliters of overnight culture were pelleted at 13,000 rpm for 5 min and used for extraction. Plasmids were eluted in EB buffer (Qiagen) and quantified on a NanoDrop Microvolume Spectrophotometer (ThermoFisher). Plasmids were further purified by running on a 0.7% agarose gel, measured against Quick-Load^®^ Purple 1 kb Plus DNA Ladder (catalog #N0550S, New England Biolabs), and extracted from the gel with a Zymoclean Gel DNA Recovery Kit (Catalog #11-300, Genesee Scientific, San Diego, CA, United States). To synthesize unmethylated plasmids, methylated plasmids were first extracted from *E. coli* DH5α and transformed into *E. coli* GM48 by heat shock in CaCl_2_ (Sambrook et al., 1989). Cells were spread on LB plates with chloramphenicol and incubated overnight, then cultured in liquid LB media with chloramphenicol and re-extracted as above.

*F. alni* DNA extraction was carried out with a CTAB protocol (Feil et al., 2012). Briefly, cells were pelleted at 10,000 rpm for 5 min in a tabletop centrifuge. Cells were then re-suspended in TE buffer and lysed with lysozyme, SDS, Proteinase K, and CTAB. DNA was extracted with 24:1 chloroform:isoamyl alcohol, and 25:24:1 phenol:chloroform:isoamyl alcohol. Extracts were then precipitated in isopropanol followed by washing in ethanol, resuspended in 170 μl DNase-free water, treated with 2 μl 100 mg/ml RNase A for 1 h (catalog #10109169001, Roche Diagnostics, Mannheim, Germany), air-dried overnight at 37°C, resuspended in 50 μl TE buffer, and quantified by NanoDrop.

### Plasmid Synthesis

A plasmid designated as pIGSAF (Supplementary Figure S1A) was designed for constitutive expression of *egfp* and synthesized by ligating a PCR-amplified fragment containing the *egfp* gene and promoter from plasmid pDiGc (Helaine et al., 2010, Addgene, Cambridge, MA, United States) to plasmid pSA3 (Dao and Ferretti, 1985). Plasmid pSA3 is a broad host-range replicating plasmid originally developed as a shuttle vector by ligating pIP501-derivative pGB305 to the *E. coli*-specific replicating plasmid pACYC184 (Dao and Ferretti, 1985). The pIP501 origin of replication has been shown to replicate in bacteria of diverse phyla including Firmicutes, from which it was originally isolated (Horodniceanu et al., 1976), as well as Actinobacteria and Proteobacteria (Kurenbach et al., 2003). Plasmid pSA3 contains chloramphenicol and tetracycline resistance genes and an *E. coli*-specific p15A origin of replication for higher-copy number propagation (Dao and Ferretti, 1985).

To synthesize PCR products for ligation, primers were designed using NCBI Primer-BLAST with default settings (Ye et al., 2012). All primers used in this study are listed in Table 1. For cloning, primers were designed with linkers adding target restriction sites onto their 5′ ends (Table 1) as well as 4–6 additional bases to aid in restriction digestion of the ends. For the synthesis of pIGSAF, primers were designed targeting the *egfp* coding region as well as the promoter region 200 base pairs upstream, amplifying a fragment 1238 base pairs in length. PCR was performed in a Bio-Rad S1000 Thermal Cycler (Bio-Rad, Hercules, CA, United States) using a Qiagen Taq PCR kit (catalog #201223). Products were synthesized by first performing 10 cycles of amplification using the lower annealing temperature corresponding to the binding site on the target DNA and then an additional 30 cycles using the annealing temperature of the full primer including the linker (Table 1).

**TABLE 1:** Primers designed for this study.

			**Melting temperatures (°C)**	
**Primer name**	**Linker restriction site**	**Sequence**	**Full primer**	**Without linker**
pSA3_Cm_F	–	TATTCAGGCGTAGCACCAGG	60.5	–
pSA3_Cm_R	–	TGTTGATACCGGGAAGCCCT	60.5	–
EGFP_*Sal*I_F	*Sal*I	TAA**GTCGAC**GAGCCGAAGCATAAACAGCG	71.9	60.5
EGFP_*Bam*HI_R	*Bam*HI	ATTAA**GGATCC**GTACCGGCATAACCAAGCCT	72.1	60.5
nif_promoter_*Xba*I_F	*Xba*I	AAGTT**TCTAGA**GTGCTCCTATTCGTTCGGC	70.8	59.5
nif_promoter_*Eco*RI_R	*Eco*RI	AAGAA**GAATTC**TTGCTCCGGGACTGAAGAC	70.8	59.5
GFP_CDS_*Eco*RI_F	*Eco*RI	AAGCT**GAATTC**ATGAGTAAAGGAGAAGAAC	66.7	50.9
GFP_CDS_*Sal*I_R	*Sal*I	AATTC**GTCGAC**TGCCTGACTGCGTTAGCAA	72.1	57.5
rpoD_qPCR_F	–	ATGCTGTTCCTGGACCTCATC	61.2	–
rpoD_qPCR_R	–	GTGGCGTAGGTCGAGAACTT	60.5	–
nifH_qPCR_F	–	GCGTACTTCAGGATGCCTCG	62.5	–
nifH_qPCR_R	–	GACGTTGTGTGTGGTGGGTT	60.5	–
infC_qPCR_F	–	ACGACGTGACCCTTCTTGGT	60.5	–
infC_qPCR_R	–	TCGGGAAGCTCGGAAGAAC	59.5	–
gfp_qPCR_F	–	TGCTTTGCGAGATACCCAGA	58.4	–
gfp_qPCR_R	–	ACGTGTCTTGTAGTTCCCGTC	61.2	–

**Restriction sites added to primers by linker PCR for use in cloning are bolded in the primer sequence and annealing temperatures are listed both for the initial reaction (“Without Linker”) and the main amplification phase of the PCR (“Full Sequence”).**

The *egfp* fragment was synthesized with *Sal*I and *Bam*HI restriction sites on the 5′ and 3′ ends, respectively, with primers EGFP_*Sal*I_F and EGFP_*Bam*HI_R. The PCR product and plasmid pSA3 were then digested with both enzymes. Digestion with *Sal*I and *Bam*HI removed a fragment approximately 200 base pairs in size from plasmid pSA3. The remaining 10 kb fragment was purified on a 0.7% agarose gel and extracted. This fragment was then mixed together with the *egfp* PCR product, denatured by heating for 5 min at 65°C, cooled on ice to allow binding of base-paired overhangs, and then the fragments were ligated together by incubation with T4 ligase (catalog #M0202S, Qiagen) at 16°C overnight. The resulting ligation was transformed into *E. coli* DH5α by heat shock (Sambrook et al., 1989). Transformants were selected on LB plates with chloramphenicol at a final concentration of 25 μg/ml. Transformed colonies were inoculated into liquid LB media with chloramphenicol and cultured as described above. Plasmid pIGSAF was then re-extracted from transformed *E. coli* and its composition was confirmed by digestion with *Sal*I and *Bam*HI. The total length of the ligated plasmid was confirmed to be approximately 12 kb by gel electrophoresis.

To study the differential expression of *egfp* in response to nitrogen limitation, a second plasmid, pIGSAFnif, was synthesized that permitted the *egfp* coding-region to be expressed under the control of the *nif* cluster promoter of *F. alni* ACN14a (Supplementary Figure S1A). The *nif* cluster promoter is upstream of the *nif* genes coding for the subunits of nitrogenase. Nitrogenase activity has been demonstrated in vesicle fractions of *Frankia* cultures but not in the hyphal fraction (Noridge and Benson, 1986; Tisa and Ensign, 1987), suggesting that *egfp* expression (GFP fluorescence) under control of the *nif* cluster promoter will be up-regulated in vesicles relative to hyphae. The *egfp* coding region of pDiGc was amplified without the upstream promoter region using primers GFP_CDS_*Eco*RI_F and GFP_CDS_*Sal*I_R (Table 1). These primers added an *Eco*RI restriction site before the start codon and a *Sal*I site 200 bases downstream of the stop codon. Separately, the 344 bases upstream of the *nif* nitrogenase cluster in the *F. alni* ACN14a genome were amplified with an *Xba*I site upstream and an *Eco*RI site downstream using primers nif_promoter_*Xba*I_F and nif_promoter_*Eco*RI_R (Table 1). These two PCR products were digested with *Eco*RI (catalog #R0101S, New England Biolabs) and ligated together as above to produce a fragment 1134 bp in length. The ligation product was then re-amplified by PCR using the *nif* promoter forward primer and the *egfp* reverse primer. The amplified ligation product and plasmid pSA3 were then each digested with *Sal*I and *Xba*I, ligated together, transformed, and selected on chloramphenicol as above. The final size of plasmid pIGSAFnif was approximately 10.8 kb (Supplementary Figure S1B).

### Frankia Transformation

*F. alni* cells were grown in culture for 1 week prior to transformation. Hyphae equivalent to approximately 50 μl packed-cell volume were collected then pelleted as above and re-suspended in 500 μl of ice-cold sterile deionized (DI) water. This wash step was repeated two more times, but after the third round of centrifugation the pellet was re-suspended in 300 μl ice-cold sterile 10% glycerol instead. Hyphae in the cell suspension were then homogenized by passage through a 21G needle twice.

Three hundred microliter of the *F. alni* cell suspension was pipetted into an electroporation cuvette with a 2 mm gap (Molecular BioProducts Catalog #5520, San Diego, CA, United States) and mixed with 10 μg of plasmid DNA. Cells were electroporated with unmethylated plasmid prepared as described above, or with methylated plasmid extracted from *E. coli* DH5α as a control. The cuvette was then incubated on ice for 5 min. Electroporation was carried out in a Bio-Rad Gene Pulser^TM^ with Pulse Controller at 2.5 kV, 200 Ω resistance, and 25 μF of capacitance. The cuvette was then immediately filled with 1 ml of ice-cold BAPP media. The cuvette was sealed with Parafilm® (Pechiney Plastic Packaging, Menasha, WI, United States) and incubated overnight without shaking at 28°C.

The following day the *F. alni* culture was removed from the cuvette and added to 3.5 ml of sterile BAPP media with tetracycline to prevent contamination, but without chloramphenicol, as above, in a glass 25 ml test tube. The culture was incubated at 28°C without shaking until visible hyphae were observed (approximately 10 days after electroporation). At this point *F. alni* hyphae were homogenized and sub-cultured into fresh media as above. Subculturing was repeated once more 1 week later. The following week (2 weeks after visible hyphae were observed) the hyphae were sub-cultured again into BAPP media, this time with chloramphenicol added for selection to a final concentration of 25 μg/ml. Chloramphenicol was chosen as the selective antibiotic because all *Frankia* strains tested by Tisa et al. (1998) were susceptible to it. This process was repeated the following week for an additional round of selection. *F. alni* cells transformed with the methylated plasmid were similarly cultured.

The presence of plasmid pIGSAF electroporated into *F. alni* cultures was then confirmed by two methods: (1) visualizing total DNA extracts from wild-type and transformed ACN14a on gels and (2) with PCR. For DNA extract visualization, 1 μg each of DNA extract from transformed and untransformed *F. alni* cultures was loaded into a well on a 0.7% agarose gel. Purified plasmid pIGSAF, extracted with a Qiaprep kit, was loaded into a separate well for comparison. Electrophoresis was run at 80 V and then the gel was visualized with a UVP High Performance Ultraviolet Transilluminator (Analytik Jena, Jena, Germany). Genomic DNA was also amplified with primer pairs pSA3_Cm_F/pSA3_CmR (Table 1) for the chloramphenicol resistance gene of plasmid pSA3 and gfp_qPCR_F/gfp_qPCR_R (Table 1) for the *egfp* gene of plasmid pIGSAF. Primers nifH_qPCR_F/nifH_qPCR_R (Table 1) for the *nifH* nitrogenase gene from the *F. alni* genome were used as a positive control for the quality of DNA extracts. Additionally, purified plasmid pDiGc from *E. coli* DH5α was used as a DNA template for *egfp* amplification as a positive control. PCR products were separated on an agarose gel and extracted as in Plasmid Synthesis, above, and sequenced by the UCDNA Sequencing Facility (University of California, Davis, CA, United States). Forward and reverse sequences were combined to make full-length amplicon sequences. These were aligned with MUSCLE (Edgar, 2004) with published reference sequences for the *egfp* and *camR* genes. Additionally, the sequences were compared by BLAST against gene sequences obtained from the original plasmids (Supplementary Figure S2).

### Confocal Microscopy

Confocal microscopy was used to visualize expression of *egfp* in *F. alni* hyphae and vesicles. *egfp* was expressed both constitutively, from plasmid pIGSAF, and differentially between (+)N (nitrogen replete) and (-)N (nitrogen limited) media under control of the *F. alni nif* promoter using plasmid pIGSAFnif. Cultures were imaged after on1 week of incubation from the previous transfer, 25 weeks after transformation, as described in Plasmid Maintenance on Selective Media, below. For imaging, *F. alni* cultures were grown either in (+)N or (–)N BAPP medium as in Culture Conditions, above, and immobilized on glass slides with a drop of 3% molten agarose solution (catalog #A9539, Sigma, St. Louis, MO, United States) maintained in a water bath at 50°C. Slides were pre-heated on a slide warmer (Fisher) at 50°C. Fifteen microliter of *Frankia* hyphal suspension was then pipetted onto each slide and covered with 35 μl of 3% molten agarose. A #1.5 coverslip was added to the *Frankia* cells in agarose, and then the slide was allowed to cool to room temperature. The *Frankia* preparations were visualized on a Leica TCS SP8 STED 3X confocal microscope with either a 20X objective, or a 100X oil-immersion objective, using either brightfield or fluorescence with a HyD detector, in the Advanced Imaging Facility, University of California, Davis. For fluorescence imaging, samples were excited with 488 nm light. The emission wavelengths were collected from 500 to 550 nm. Images were stored as .lif files from Leica LAS X and then viewed in FIJI (Schindelin et al., 2012). For imaging of individual hyphae and vesicles, the 100X objective lens was used and images were taken in Z-stacks with a step size of 0.1 μm. To visualize *F. alni* colonies at low magnification, Z-stack images were taken with the 20X objective in five steps of 2 μm each and then combined using the highest fluorescent intensity of each pixel (FIJI MAX setting).

### qPCR Verification of Differential Gene Expression

Quantification of gene expression and fold-changes were obtained based on qPCR amplification as an additional line of evidence demonstrating the plasmid transformation and the differential expression of genes (hyphae vs. vesicles) regulated by the *nif* promoter under nitrogen-fixing conditions. Cultures of transformed cells were grown in 4.0 ml (+)N and (–)N media in sterile six-well plates with shaking at 50 rpm at 28°C. After 5 days, RNA was extracted by bead-beating, following a protocol adapted from Dietrich et al. (2000): *F. alni* hyphae were pelleted at 9000 rpm for 15 min, resuspended in 1050 μl Buffer RLT (Qiagen), and transferred to 2 ml tubes containing Lysing Matrix B (catalog #6911-100, MP Biomedicals, Burlingame, CA, United States). Samples were processed with a FastPrep FP120 (Thermo Fisher Scientific, Waltham, MA, United States) for 45 s at setting 6.5, then placed on ice for 45 s. The processing step was then repeated twice more with cooling on ice between each step. Supernatants were then transferred to Qiagen RNeasy spin columns and purified with a Qiagen RNeasy Mini Kit (catalog #74104). RNA was eluted in RNase-free water and then contaminating DNA was digested with an Invitrogen TURBO DNA-free Kit (catalog #AM1907, Waltham, MA, United States). Finally, cDNA was synthesized with an Invitrogen Superscript III Kit (catalog #18080051) with random hexamer primers. qPCR was performed on a 7500 Fast Real-Time PCR System (Applied Biosystems, Foster City, CA, United States) with Fast SYBR Green qPCR Master Mix (catalog #4385612, Applied Biosystems) and primer pairs rpoD_qPCR_F/rpoD_qPCR_R, nifH_qpCR_F/nifH_qpCR_R, infC_qPCR_F/infC_qPCR_R, and gfp_qPCR_F/gfp_qPCR_R (Table 1). One microliter of cDNA was used in each reaction. The *egfp* gene from plasmid pIGSAF and *nifH* (FRAAL6813) gene from the *F. alni* genome were used as experimental targets to confirm differential regulation of *egfp* under nitrogen limitation. Housekeeping gene *infC* (FRAAL5216) was used for normalization as in Alloisio et al. (2010) and sigma factor gene *rpoD* (FRAAL2026) was used as a negative control. To quantify fold-change the ΔΔCt values for each sample were calculated then converted into fold-changes equal to 2^–ΔΔ*Ct*^ (Livak and Schmittgen, 2001).

### Plasmid Copy Number

The copy number of plasmid pIGSAF in *F. alni* cultures under antibiotic selection was determined with both the absolute and relative quantification methods of Lee et al. (2006). For absolute quantification standards were created by serial 10-fold dilution, repeated five times, of the plasmid pDiGc extracted from *E. coli* DH5α and *F. alni* genomic DNA from an untransformed culture. These standards were quantified with qPCR using primer pair gfp_qPCR_F/gfp_qPCR_R and nifH_qPCR_F/nifH_qPCR_R respectively (Table 1). One microliter of DNA extract was used for each reaction. Standard curves were created by linear regression fit to a plot of log copy number vs. qPCR cycles (Ct) of the standards, resulting in strong correlations (*R*^2^ = 0.99) for both curves. The log number of copies of the *egfp* gene of plasmid pIGSAF per sample was calculated with the Ct value of amplification by primer pair gfp_qPCR_F/gfp_qPCR_R (Table 1) and the regression equation of the standard curve and then back-transformed. The number of copies of the *infC* and *nifH* genes of the *F. alni* genome (FRAAL5216) were calculated the same way with primer pairs infC_qPCR_F/infC_qPCR_R and nifH_qPCR_F/nifH_qPCR_R (Table 1). The resulting copy numbers of the plasmid-bound *egfp* gene and genome-bound *infC* (FRAAL5216) and *nifH* (FRAAL6813) genes per sample were then used to calculate the ratio of plasmid to genome. qPCR reactions were performed in duplicate on DNA extracts from each of three separate cultures and then the copy numbers were averaged with a geometric mean.

For relative quantification, the same DNA extracts as above were used and the *F. alni nifH* (FRAAL6813) gene was amplified with primer pair nifH_qpCR_F/nifH_qpCR_R (Table 1) as well. The ΔΔCt method of Lee et al. (2006) was used to estimate the difference in fold-change between *egfp* (on the plasmid) and *nifH* (on the genome, FRAAL6813) between the samples, using *infC* (FRAAL5216) quantification as the endogenous control for normalization. The ΔΔCt values for each sample were calculated as in qPCR Verification of Differential Gene Expression, above, and then averaged with a geometric mean.

### Plasmid Maintenance on Selective Media

Three separate transformation experiments were carried out, from August 2017 through March 2019, as outlined in Table 2. Transformed cultures were maintained with weekly subculturing into fresh selective media and used in experiments over the course of several months following each transformation. Initial transformations of *F. alni* with plasmid pIGSAF were performed in August of 2017; the cultures were maintained by subculturing and were visualized by confocal microscopy from December 2017 (17 weeks after transformation) through March 2018 (32 weeks). Transformation of *F. alni* with plasmid pIGSAFnif was performed in January 2018, selected as above, and imaged by confocal microscopy in April 2018 (15 weeks). These cultures were maintained by subculturing in selective media for RNA extraction for qPCR through July 2018 (27 weeks). A third round of transformants was created for use in the Plasmid Persistence assay detailed below. These cultures were transformed in September 2018 and subcultured weekly in selective media through March 2019. The presence of the plasmid was confirmed by DNA extraction and PCR amplification of the *egfp* gene in March 2019 (25 weeks) as described in *Frankia* Transformation, above.

**TABLE 2:** Separate transformations performed on *Frankia alni* ACN14a and cultures maintained on selective media.

**Culture**	**Date performed**	**Period maintained in selective media with routine subculture**	**Plasmid**	**Role**	**Experiments performed**
1	August 2017	7 months	pIGSAF	Constitutive *egfp*	PCR verification of plasmid and confocal microscopy
2	January 2018	6 months	pIGSAF nif	*nif-*Promoter Regulated *egfp*	qPCR verification of differential expression and confocal microscopy
3	September 2018	7 months (to date)	pIGSAF	Constitutive *egfp*	Stability in non-selective media, copy number calculation, confocal microscopy, DNA extract, and PCR verification

**For each transformation the plasmid used and the experiments performed are given.**

### Plasmid Persistence in Non-selective Media

To test the persistence of plasmid pIGSAF in transformed *F. alni* without selection, a time-course of growth in media without antibiotics was performed. Fresh cultures were transformed with plasmid pIGSAF and initially selected as described in *Frankia* Transformation, above. After this 4-week selection process, cultures were then grown in non-selective media: cells were first pelleted and re-suspended in fresh BAPP media, then subcultured without the addition of chloramphenicol into six-well plates and incubated for 1 week. A separate set of cultures from the same stock was maintained in selective media and subcultured at the same time points as a control. Each week both sets of cultures were pelleted and re-suspended in 500 μl fresh BAPP media. The suspensions were homogenized by passage through a 21G needle twice. 250 μl of each homogenate was transferred to 4 ml fresh BAPP media without chloramphenicol in each well and the remaining 250 μl was used for total genomic DNA extraction as described in DNA Extraction above. This process was repeated once per week for 4 weeks. At each sampling point, the relative amount of plasmid in each sample was quantified by qPCR, performed in duplicate for each of three biological replicates, using *egfp* primers GFP_qPCR_F and GFP_qPCR_R (Table 1). Fold-change of plasmid between each time point was calculated using the ΔΔCt method (Lee et al., 2006). The *infC* gene (FRAAL5216), amplified from the same DNA extracts with primers infC_qPCR_F and infC_qPCR_R (Table 1), was used as a control to normalize the amount of DNA in each sample. To determine significant changes in plasmid abundance, two-tailed Welch’s *t*-tests were performed in R on normalized ΔCt values (*p* < 0.05). Cultures grown with and without selection for 4 weeks were also imaged with fluorescence according to methods in Confocal Microscopy, above.

## Results

### Identification of Restriction Enzymes in Frankia and Transcriptome Analysis

**FIGURE 1:**
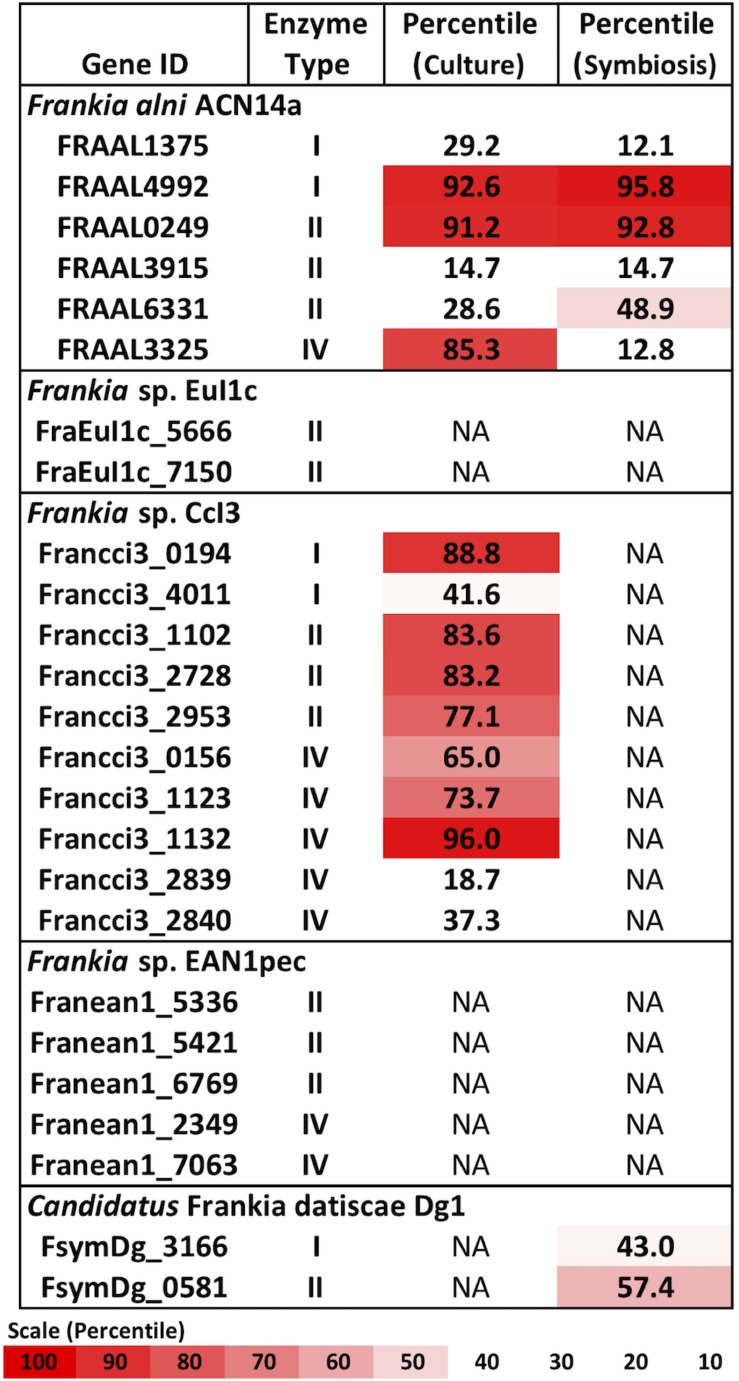
Restriction enzymes annotated in the *Frankia* genomes and expression levels of each gene in the transcriptome in culture when available (Alloisio et al., 2010; Bickhart and Benson, 2011). NA: Transcriptome not available.

Types I, II, and IV restriction enzymes were identified in several *Frankia* genomes (Figure 1). Examining the transcriptome of *Frankia alni* ACN14a grown in (+)N culture (Alloisio et al., 2010), we found that three restriction enzyme genes were highly expressed: one type I enzyme (FRAAL4992, 92nd percentile), one type II enzyme (FRAAL0249, 91st percentile) and one type IV enzyme (FRAAL3325, 85th percentile) (Figure 1). The type IV enzyme was annotated in REBASE as a “Type IV Methyl-directed restriction enzyme” of the Mrr methyladenine-targeting family. Other *Frankia* genomes contained significant homologs of this type IV enzyme as well. The genome of *Frankia casuarinae* CcI3 contained five type IV enzymes, the most of any *Frankia* genome examined (Figure 1). In (+)N and (-)N-grown transcriptomes of *Frankia* sp. CcI3, type IV Mrr enzymes were very highly expressed, up to the 95th percentile (Figure 1). One of the five type IV enzymes in CcI3 (Francci3_2839) was annotated as a Mcr type IV enzyme, which targets 5-methylcytosine instead of methyladenine (REBASE) and its expression was very low, in only the 18th percentile of genes in the transcriptome. Of the five complete genomes examined, only the cluster II *Frankia Candidatus* Frankia datiscae Dg1 did not contain any putative type IV restriction genes.

A symbiotic transcriptome of *F. alni* ACN14a from root nodules is available in addition to transcriptomes of free-living (nitrogen-replete) culture (Alloisio et al., 2010). In symbiosis with *Alnus glutinosa* the *mrr* homolog was down-regulated approximately 7.8-fold (*p* < 0.007), from the 85th percentile in culture to an expression level no higher than 12% of transcriptome genes, while the other restriction enzymes identified in the *F. alni* genome (type I and type II) were not down-regulated in symbiosis (Figure 1). The other available *Frankia* symbiotic transcriptome, *Frankia* sp. Dg1 (Persson et al., 2015), showed very low expression of its two restriction enzymes (type I and type II, Figure 1).

**FIGURE 2:**
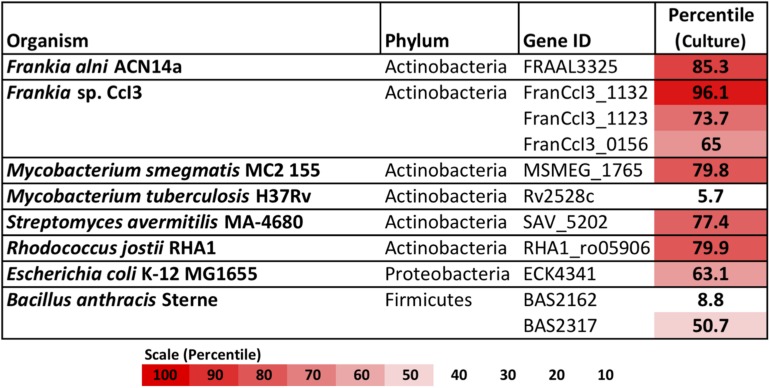
Restriction enzymes annotated in the genomes of *Frankia*, with other actinobacteria, proteobacteria, and firmicutes for comparison. For each, expression levels for available transcriptomes growing in pure culture are provided.

Of the complete actinobacterial genomes outside of genus *Frankia* with published transcriptomes that were analyzed, *Mycobacterium smegmatis*, *Streptomyces avermitilis*, and *Rhodococcus jostii* also highly expressed type IV restriction enzyme genes in culture, in the upper-70th percentiles of their respective transcriptomes (Figure 2). In comparison, the transcriptomes of proteobacteria and firmicutes examined expressed their corresponding *mrr* genes around the 50th or 60th percentiles. One notable exception was the actinobacterium *Mycobacterium tuberculosis*, which expressed its single gene for a methyladenine-directed restriction enzyme (Mrr) at extremely low levels, around the 6th percentile in culture.

### Genetic Transformation of *Frankia alni* ACN14a With an Unmethylated Replicating Plasmid

**FIGURE 3:**
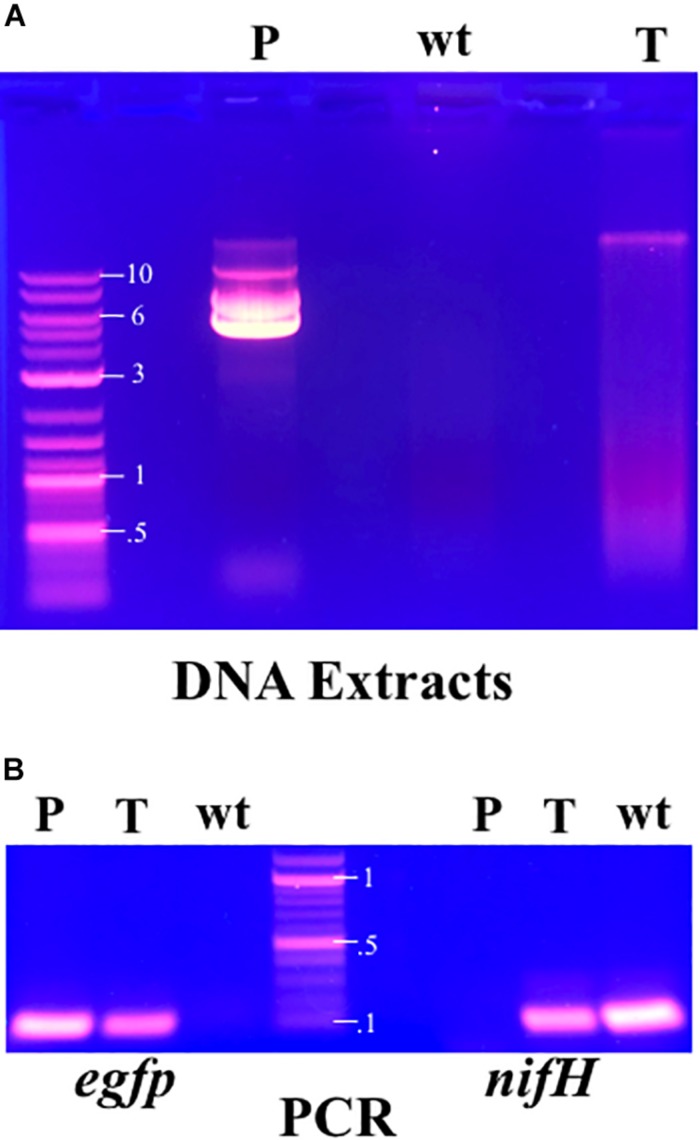
**(A)** Total DNA extracts from cultures of transformed and untransformed *F. alni* maintained in culture with routine subculture for 25 weeks from transformation, analyzed by gel electrophoresis. The extract from transformed *F. alni* (T, right) shows an additional band not present in untransformed, wild-type, cells (wt, middle) equivalent to linearized plasmid pIGSAF from *E. coli* (P, left). **(B)** PCR amplification of *egfp* (left, 120 bp) and *nifH* (right, 131 bp) genes. Amplification was performed on purified plasmid pDiGc (P, left) and genomic DNA extracts from *F. alni* transformed with plasmid pIGSAF (T) and wild-type *F. alni* (wt), respectively. Labels on DNA ladder denote sizes in kilobases (kb). The plasmid carrying *egfp* is not present in the wt *F. alni* DNA and the *nifH* gene is not present in purified plasmid pDiGc.

After electroporation *F. alni* cells formed visible hyphae in culture after about 10 days. When subcultured into chloramphenicol-selective media, *F. alni* cultures transformed with unmethylated plasmid pIGSAF were able to grow, whereas untransformed cultures and those transformed with methylated plasmid were not. When analyzed on the gel, purified plasmid pIGSAF from *E. coli* formed bands representing linear (approximately 12 kb), circular (10 kb), and supercoiled (6 kb) plasmid (Figure 3A). Presence of plasmid pIGSAF in transformed cultures was verified by a band present in DNA extracts corresponding to the linear form of plasmid pIGSAF (calculated size 11.8 kb) that was not present in extracts from wild-type *F. alni* (Figure 3A). PCR amplification and sequencing of the *egfp* gene from DNA extracts of transformed *F. alni* cultures confirmed the identity of the plasmid (Figure 3B and Supplementary Figure S2). Absolute copy number quantification with qPCR estimated the plasmid was present at 12.5 copies of plasmid per molecule of genomic DNA; relative quantification gave a similar estimate of 11.8 copies per genome (Supplementary Table S2). Each experiment was performed on cultures maintained with routine weekly subculture for 15–32 weeks post-transformation, as described in the Methods.

**FIGURE 4:**
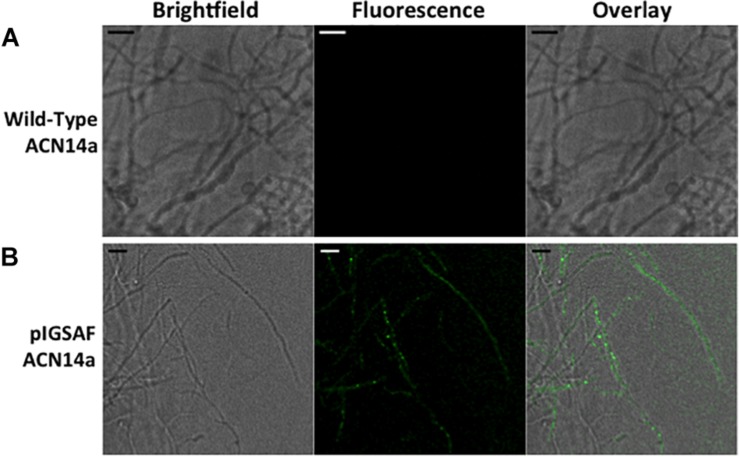
Transformation of *Frankia alni* ACN14a with a plasmid expressing *egfp*. **(A)** Wild-type *F. alni* ACN14a control. **(B)** Transformed *F. alni* ACN14a with plasmid pIGSAF. Images were obtained on a confocal microscope with a 100X objective with both brightfield and fluorescence and then overlaid. Size bars equal 5 μm.

When imaged under 488 nm wavelength of excitation in the confocal microscope, green fluorescence typical of GFP was observed in hyphae as shown in Figure 4. Wild-type *F. alni* hyphae displayed autofluorescence around 575 nm as observed by Hahn et al. (1993), but no autofluorescence was observed in the 500–550 nm range (Figure 4).

### Differential Regulation of *egfp* Under the Control of the *Frankia alni* ACN14a *nif* Cluster Promoter

When grown in (–)N culture, transformants carrying the pIGSAFnif plasmid showed significant up-regulation of the *egfp* gene conjugated to the *nif* cluster promoter, approximately 100-fold relative to (+)N culture, or approximately 8.5-fold per copy of plasmid (Table 3). The *F. alni nifH* gene (FRAAL6813), used as a positive control for nitrogen fixation, was similarly significantly up-regulated approximately 8.5-fold in (–)N media compared with (+)N cultures. Expression of the *F. alni rpoD* housekeeping gene (FRAAL2026), used as a negative control, was not significantly different between (+)N and (–)N cultures (Table 3).

**TABLE 3:** qPCR verification of differential regulation of *egfp* in (+)N and (–)N media.

**Gene**	**Role**	**Fold change (-N/ + N)**	**Copy number**	**Fold change per copy**	***p*-value**
*nif:egfp*	Fluorescence	101.6	12	8.5	<0.05
*nifH*	Nitrogen fixation	8.5	1	8.5	<0.05
*rpoD*	Housekeeping	0.4	1	0.4	>0.05

**qPCR tested fold-change of egfp as well as nifH as a nitrogen-fixation positive control and rpoD as a housekeeping negative control.**

**FIGURE 5:**
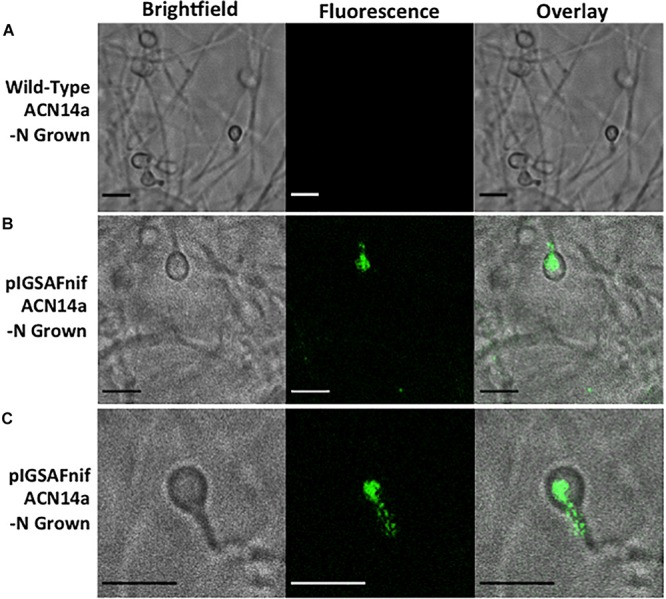
Fluorescence detection of transformation of *Frankia alni* ACN14a with a plasmid expressing *egfp* under the control of the *nif* cluster promoter region from the *F. alni* genome. Transformed cultures were grown in both (+)N and (–)N media to compare fluorescence with and without nitrogen fixation. **(A)** *F. alni* ACN14a transformed with plasmid pIGSAFnif grown in (+)N media. **(B)** *F. alni* ACN14a with plasmid pIGSAFnif grown in (–)N media. **(C)** Image of a vesicle showing fluorescence in the stalk. Images were obtained on a confocal microscope at 100X magnification with brightfield illumination and fluorescence imaging and then overlaid. Size bars equal 5 μm.

*F. alni* containing pIGSAFnif grown in (–)N media fluoresced predominantly in the vesicles, observed at 100X magnification (Figure 5 and Supplementary Figure S3). Little to no fluorescence was observed in hyphae. Fluorescence in the vesicles was present both in the spherical portion as well as in the stalk connecting the vesicle to the hyphae. No fluorescence was observed in hyphae or vesicles of wild-type *F. alni* grown in (–)N media (Figure 5A). Due to the step size of 0.1 μm bright fluorescence was only observed when vesicles were in the plane of focus (Supplementary Figure S3). Few vesicles were present in images likely since the cells were observed after 1 week of culture in (-)N medium.

### Stability of Plasmid pIGSAF in *F. alni* ACN14a in the Absence of Selection

**FIGURE 6:**
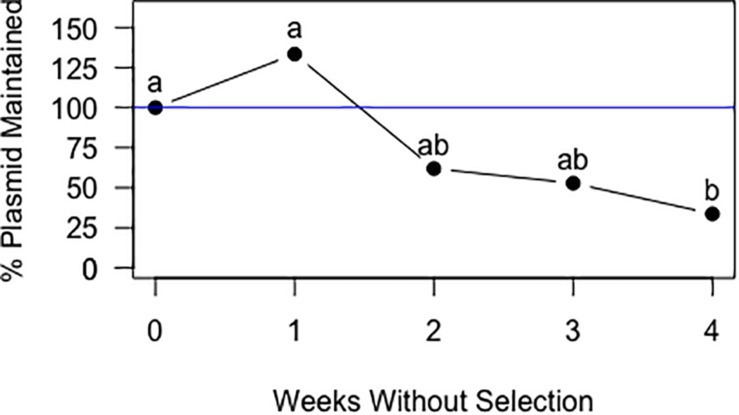
Maintenance of plasmid pIGSAF in *Frankia alni* in culture without chloramphenicol selection over the course of 4 weeks. Each week the culture was sub-cultured into fresh media and genomic DNA was extracted to measure relative plasmid concentrations via qPCR. Labels “a” and “b” indicate time points that are significantly different from each other (*p* < 0.05).

**FIGURE 7:**
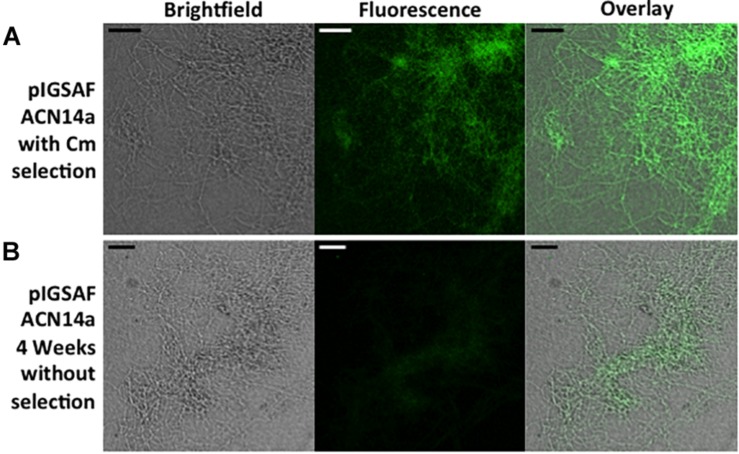
Fluorescence of a *Frankia alni* hyphal colony transformed with plasmid pIGSAF grown **(A)** with chloramphicol selection for 10 weeks **(B)** and without selection for 4 weeks. Colonies were viewed at 20X magnification. Size bars equal 25 μm.

In cultures grown without chloramphenicol selection, statistical analysis of the qPCR data for *Frankia* cells transformed with pIGSAF did not show a significant difference in the amount of plasmid relative to genomic DNA after one, two, and three rounds of sub-culturing (Figure 6). Only after the fourth round of sub-culturing was a significant decrease from the initial plasmid concentration detected by qPCR (Figure 6), however, even after 4 weeks without selection, fluorescence throughout the colony was still readily observed (Figure 7B).

## Discussion

### Unmethylated DNA Circumvents the Restriction Barrier to Genetic Transformation of *Frankia alni* ACN14a With a Replicating Plasmid

We have shown that *F. alni* can be stably transformed with an unmethylated replicating plasmid introduced by electroporation. The methods presented here circumvent two major transformation barriers in *Frankia*. First, the lack of methylation avoids restriction of the plasmid by type IV methyl-directed restriction enzymes. Second, the use of a plasmid replicated and maintained outside the genome does not rely on the reduced homologous recombination rate in actinobacteria (Zhang et al., 2012). Plasmids pIGSAF and pIGSAFnif (Supplementary Figure S1) were stably maintained in *F. alni* culture and used to perform routine experiments. Three independent transformations were performed over the course of 2 years and the resulting transformants were maintained on selective media by repeated subculture for at least 7 months (Table 2). The presence and stability of the plasmids were confirmed with gels of whole genome DNA extracts (Figure 3A), PCR and qPCR amplification of plasmid-bound genes (Figure 3B, Table 3, and Supplementary Figure S2), and visualization of GFP expressed from plasmids (Figures 4, 5, 7). The copy number of plasmid pIGSAF per genome in *F. alni* was determined to be about 12 copies per cell (Table 3), in line with findings for other plasmids derived from plasmid pIP501 that have been estimated to be maintained at approximately 10 copies per cell (Behnke et al., 1981). In addition, plasmid pIGSAF was determined to be stable in non-selective media for a period of at least 3 weeks (Figure 6).

Of the restriction enzymes identified in *Frankia* genomes (Figure 1) our analysis indicated that the type IV enzymes posed the most likely barrier to transformation. Types I and II enzymes recognize specific sequence motifs of generally six to eight nucleotides (Tock and Dryden, 2005) and hence are statistically highly unlikely to be a broad-range barrier to transformation or horizontal transfer (Thomas and Nielsen, 2005). Additionally, in the genome of *F. alni* ACN14a the majority of types I and II genes showed very low expression in both (+)N-culture and symbiosis (Figure 1).

A type IV homolog of an *mrr* type methyladenine-targeting restriction gene was highly expressed in *F. alni* in culture (Figure 1), suggesting that DNA with methylated adenine bases is degraded in this organism. Actinobacteria, especially *Frankia*, express type IV methyl-directed restriction enzyme genes more highly in culture than do proteobacteria and firmicutes (Figure 2), a finding that correlates with previous reports of higher transformation efficiencies with unmethylated plasmids than methylated in *Corynebacterium* (Ankri et al., 1996) and *Streptomyces* spp. (Molle et al., 1999). Genomes of the majority of actinobacteria are missing homologs of the *dam* methyltransferase gene (Sanchez-Romero et al., 2015) whose product is used to mark parent DNA strands during replication, and *mutS* and *mutL* that form a complex for the removal and repair of mismatched bases on the daughter strand determined by the methylation of adenine residues (Sachadyn, 2010). Together, these factors suggest a preference for unmethylated over methylated DNA among most of the actinobacteria.

Type IV restriction enzymes have been suggested to have evolved as a counter to phage methylation systems that themselves evolved to evade host restriction systems through the methylation of restriction target sites (Westra et al., 2012). Phage genomes adopt the methylation patterns of their previous host (Loenen and Raleigh, 2014) thus increasing the likelihood of digestion by actinobacterial enzymes if replicated in a *dam* + host. The expression of type IV restriction enzymes in actinobacteria therefore could represent an adaptation to prevent infection by phages based on the methylation state of their genomes. Differences in methylation patterns between actinobacteria and other bacterial phyla (Novella et al., 1996) potentially constitute a barrier to horizontal gene transfer between these groups, including phage-mediated gene transfer.

Of particular interest to the evolution of root nodule symbioses is the possibility of transfer of relevant genes between *Frankia* and the rhizobia, and vice versa. It has been suggested that the *nodA* gene involved in Nod factor biosynthesis evolved in the actinobacteria, including some *Frankia*, and was then horizontally transferred to the rhizobia (Persson et al., 2015). If type IV restriction enzymes create a barrier to horizontal transfer into actinobacteria from *dam* + bacteria including proteobacteria, it would seem that horizontal transfer from actinobacteria to other phyla would be more likely than the reverse.

However, *F. alni* was observed to down-regulate its type IV *mrr* gene substantially in symbiosis (Figure 2). As roots contain much lower concentrations of bacteriophage than the surrounding soil (Ward and Mahler, 1982) this could represent a decreased necessity for restriction enzymes as a defense mechanism during symbiosis. A potential side-effect of this down-regulation, however, is that the barrier to horizontal transfer posed by type IV enzymes is likely lowered during symbiosis. In plants, the endophytic compartment is dominated by actinobacteria, with specific taxa of other phyla including proteobacteria and bacteroidetes (Lundberg et al., 2012). Due to the barrier created by type IV enzymes in the free-living condition (i.e., outside symbiosis), horizontal transfer involving *Frankia* in the soil is likely limited to other actinobacteria whose genomes similarly lack methylation (Novella et al., 1996), many of whom are endophytes (Trujillo et al., 2015). Inside the host, however, *Frankia* is likely more receptive to genes from other endophytic taxa as well. Combined, these factors could result in the preferential acquisition of genes involved in growth and host-microbe interactions *in planta*. In legume symbioses, exudates from hosts into their rhizospheres have been proposed to promote the conjugative transfer of symbiosis genes from a donor group of rhizobia to others, broadening the range of symbionts available to the host (Ling et al., 2016). Down-regulation of type IV restriction genes in actinorhizal nodule symbioses could be another mechanism that enhances horizontal transfer of genes related to interactions with plants by making the recipient *Frankia* more susceptible in the endophytic environment.

*M. tuberculosis* showed much lower transcription of its annotated type IV methyladenine targeting restriction enzyme than other actinobacteria. *M. tuberculosis* expresses an adenine methyltransferase in hypoxic conditions that regulates the expression of genes likely involved with survival during macrophage infection (Shell et al., 2013). For this reason it is likely that *M. tuberculosis* responds to methylated DNA differently than other actinobacteria; indeed electrotransformation of *M. tuberculosis* can be readily achieved with methylated plasmids replicated in *E. coli* DH5α (Pelicic et al., 1997), suggesting that methylated DNA is not digested in *M. tuberculosis*.

In this study derivatives of broad host-range plasmid pSA3 were capable of replication in *F. alni*. This shows that the broad host-range origin is capable of replication in *Frankia* and supports its use as a vector for the manipulation of *Frankia* spp. The parent plasmid of pSA3, pIP501, replicates in a very broad range of bacteria including *Streptomyces lividans* and *E. coli* (Kurenbach et al., 2003) indicating the potential for transformation of additional actinobacteria with these plasmids.

### Differential Regulation of *egfp* Under the Control of the *Frankia alni* ACN14a *nif* Cluster Promoter

The expression of the *egfp* gene of plasmid pIGSAFnif was up-regulated in (–)N media compared with expression in (+)N media, at proportional levels to the expression of the *nifH* nitrogenase gene (FRAAL6813, Table 3), demonstrating for the first time that expression of reporter genes can be manipulated in *Frankia*. This transformation system resulted in the ability to visualize the expression of nitrogen fixation genes *in vitro* by fluorescence microscopy (Figure 5). Interestingly, fluorescence was detected in both the spherical portion of the vesicle as well as in the stalk that connects to the hyphae, suggesting that nitrogen fixation genes are expressed in both parts of the vesicle. Previous studies have shown that the vesicle envelope is deposited around the stalk as well as the spherical part of the vesicle (Lancelle et al., 1985), supporting the observation that nitrogen fixation can occur in the stalk.

Although the fluorescence observed when *egfp* was expressed under the control of the *F. alni nif* cluster promoter was predominantly in the vesicles, some fluorescence was occasionally observed in hyphae under nitrogen-fixing conditions whereas in (+)N media there was no observable fluorescence (Figure 5). This suggests that there can be condition-dependent expression of *nif* genes in the hyphae as well as the vesicles induced by nitrogen limitation. *Frankia* spp. in symbiosis with members of the Casuarinaceae have been reported to fix nitrogen in hyphae, since no vesicles are differentiated (Murry et al., 1985); this pattern correlated with the formation of a lignified host cell wall in the symbiotic tissue that likely reduces oxygen partial pressure (Berg and McDowell, 1987). In liquid culture, there may be zones of low pO_2_ that develop in portions of a *Frankia* hyphal colony where nitrogen fixation could be induced.

*Frankia* spp. in symbiosis have been suggested to be more autonomous than rhizobial microsymbionts due to their ability to control the flow of oxygen with the formation of vesicles, and due as well to the expression of more metabolic pathways in the microsymbiont in symbiosis. These factors potentially allow *Frankia* to be more metabolically independent from their hosts (Alloisio et al., 2010; Berry et al., 2011). The development of genetic tools for the manipulation of *Frankia* will allow further exploration into these and other distinctive molecular aspects of actinorhizal symbioses, which will, in turn, further inform analyses of the evolution and diversity of root nodule symbiosis.

### Future Directions

The transformation methods presented here should be applicable for genetic experiments in other *Frankia* strains. Because plasmid pIGSAF has a broad host-range origin of replication and expresses *egfp* under the control of a constitutive promoter, the plasmid is likely to be usable in other strains as well. Even in the absence of selection, plasmid pIGSAF was found to be stable in *F. alni* cultures for at least 3 weeks (Figure 6) and cultures continued to show fluorescence after at least 4 weeks (Figure 7). Three to 4 weeks is a time period that sufficiently spans the stages of nodulation and early nitrogen fixation in a broad-spectrum of hosts. This includes *Alnus glutinosa* with *F. alni* ACN14a (Alloisio et al., 2010), and *Casuarina cunninghamiana* (Torrey, 1976), *Discaria trinervis* (Valverde and Wall, 1999), *Shepherdia argentaea* (Racette and Torrey, 1989), and *Datisca glomerata* (Berry et al., 2004). This suggests that transformants can be used to inoculate plants to study of the role of *Frankia* and its interactions with hosts during nodule establishment and symbiosis. In future this system could be modified using recombination or site-specific integrases to anchor genes within the genome. Differential regulation of reporter genes such as *egfp* can be used to localize the expression of genes identified by genomics and transcriptomics in specific *Frankia* cell types, in different growth conditions, and in symbiosis. Replicating plasmids may also enable the study of gene function by constitutive expression of selected genomic genes, by promoter switching or by knock-down experiments expressing anti-RNAs to genes of interest (Gillaspie et al., 2009). Circumventing the natural restriction systems of *Frankia* will also increase the transformation rate of non-replicating plasmids and enable higher efficiency recombination, which can be combined with CRISPR systems, for gene knock-out experiments as attempted by Kucho et al. (2009).

While this manuscript was in review a separate method for the transformation of *Frankia* spp. utilizing conjugation with a methylation-positive *E. coli* was reported by Pesce et al. (2019). Conjugative transfer has been shown to evade recipient restriction systems (Stein et al., 1988; Baltz, 1994), thus presumably allowing *Frankia* spp. to circumvent the type IV restriction barrier identified in this study. Nevertheless, the transformation efficiency of *Streptomyces* spp. by conjugation has been shown to increase over 10^4^-fold with unmethylated DNA (Flett et al., 1997). Thus, in addition to enabling the transformation of *Frankia* spp. by electroporation, our analysis of restriction systems in *Frankia* spp. can be further utilized to improve the transformation efficiency of conjugative transfer to *Frankia* spp. through the use of methylation-deficient *E. coli* donors. Optimized transformation efficiency will be crucial for future studies involving the generation of recombinants including gene knock-outs.

## Author Contributions

IG and AB conceived the project. IG carried out the project and wrote the manuscript. SV and GN assisted with the experiment and designed primer sequences. AB edited the manuscript.

## Conflict of Interest

The authors declare that the research was conducted in the absence of any commercial or financial relationships that could be construed as a potential conflict of interest.

## Supplementary Material

FIGURE S1 Plasmid maps of pIGSAF **(A)** and pIGSAFnif **(B)**, derived from plasmid pSA3 (Dao and Ferretti, 1985). Maps depict the *egfp* gene (green), the promoter of *egfp* (yellow), the origins of replication (blue), and the chloramphenicol resistance gene (*camR*, red). ori corresponds to the p15A *E. coli*-specific high-copy number origin originally derived from pACYC184 (Rose, 1988). Rep and Cop correspond to the broad host-range origin and copy number control regions derived from pGB305 (Dao and Ferretti, 1985), respectively. Also shown are unique restriction sites, and the *Eco*RI site introduced by ligation of the *nif* promoter to the *egfp* coding region in plasmid pIGSAFnif.

FIGURE S2 Alignments and BLAST *e*-values of *egfp* and *camR* gene PCR products amplified from transformed *F. alni* DNA extracts. PCR sequences were compared against those of published reference sequences for each gene.

FIGURE S3 100X magnification confocal images showing fluorescent vesicles in different planes of focus. Two vesicles shown are in the same confocal view as in Figure 5 **(A)**, but are more visibly fluorescent when focused **(B).** These vesicles can be seen in Figure 5, but with less fluorescence at that particular plane of focus.

TABLE S1 Listing of genomes and transcriptomes used in this study. Transcriptomes are listed with their GEO accession number and original reference.

TABLE S2 Standard curve and copy number calculations of plasmid pIGSAF determined by qPCR with relative and absolute quantification.
