# Supplementary figures and images for: A Stable Genetic Transformation System and Implications of the Type IV Restriction System in the Nitrogen-Fixing Plant Endosymbiont Frankia alni ACN14a
Source: Front Microbiol. 2019 Sep 24;10:2230. doi: 10.3389/fmicb.2019.02230 (PMC6769113; doi:10.3389/fmicb.2019.02230)

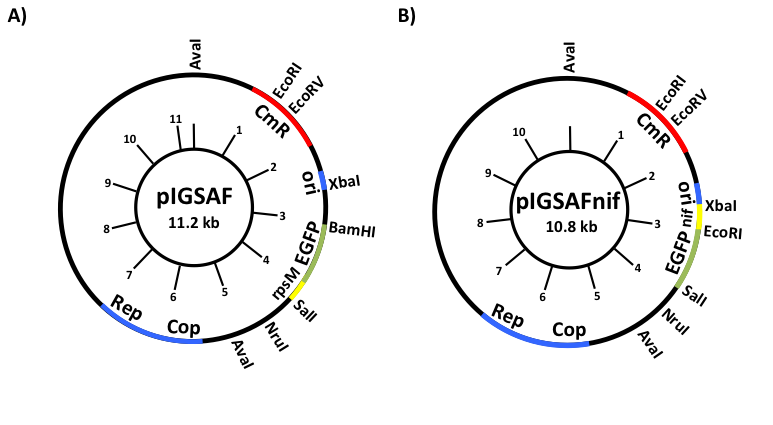

Supplement: FIGURE S1 — Plasmid maps of pIGSAF (A) and pIGSAFnif (B), derived from plasmid pSA3 (Dao and Ferretti, 1985). Maps depict the egfp gene (green), the promoter of egfp (yellow), the origins of replication (blue), and the chloramphenicol resistance gene (camR, red). ori corresponds to the p15A E. coli-specific high-copy number origin originally derived from pACYC184 (Rose, 1988). Rep and Cop correspond to the broad host-range origin and copy number control regions derived from pGB305 (Dao and Ferretti, 1985), respectively. Also shown are unique restriction sites, and the EcoRI site introduced by ligation of the nif promoter to the egfp coding region in plasmid pIGSAFnif. [file Image_1.TIF]

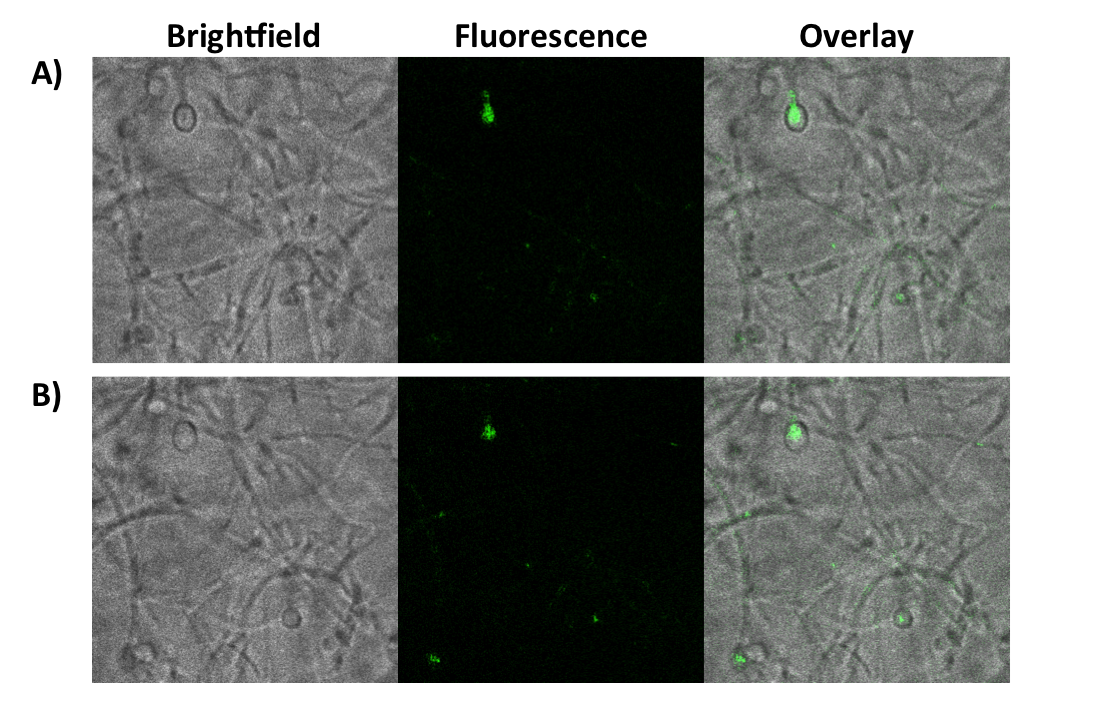

Supplement: FIGURE S3 — 100X magnification confocal images showing fluorescent vesicles in different planes of focus. Two vesicles shown are in the same confocal view as in Figure 5 (A), but are more visibly fluorescent when focused (B). These vesicles can be seen in Figure 5, but with less fluorescence at that particular plane of focus. [file Image_3.tif]
