# Supplementary material for: A Stable Genetic Transformation System and Implications of the Type IV Restriction System in the Nitrogen-Fixing Plant Endosymbiont Frankia alni ACN14a
Source: Front Microbiol. 2019 Sep 24;10:2230. doi: 10.3389/fmicb.2019.02230 (PMC6769113; doi:10.3389/fmicb.2019.02230)
Supplement: FIGURE S2 — Alignments and BLAST e-values of egfp and camR gene PCR products amplified from transformed F. alni DNA extracts. PCR sequences were compared against those of published reference sequences for each gene. [file Image_2.pdf]

egfp

Primers: GFP_qPCR_F and GFP_qPCR_R

BLAST Hit vs. pDiGc *egfp*: 7e-58

Alignment (*egfp* bases 209-331)

```
pDiGc_egfp      TGCTTTGCGAGATACCCAGATCATATGAAACAGCATGACTTTTTCAAGAGTGCCATG-CC
Falni_egfp      TGCTTTGCGAGATACCCAGATCATATGAAACAGCATGACTTTTTCAAGAGTGCCATGCCC
                ********************************************************* **

pDiGc_egfp      CGAAGGTTATGTACAGGAAAGAACTATATTTTTCAAAGATGACGGGAACTACAAGACACG
Falni_egfp      CGAAGGTTATTCACAGGAAAGAACTATATTTTTCAAAGATGACGGGAACTACAAGACACG
                **********  ************************************************

pDiGc_egfp      T
Falni_egfp      T
                *
```

camr

Primers: pSA3_Cm_F and pSA3_Cm_R

BLAST Hit vs. pSA3 *camr*: 1e-168

Alignment (camR bases 266-772)

```
Falni_camR      GCAATGAAAGACAGAGAGATGAAAATATTAGATAGTTTTCACCATGAGGTTCCAACTTTC
pSA3_camR       GCAATGAAAGACGGTGAGCTGGTGATATGGGATAGTGTTCACCCT--TGTTACACCGTTT
                ************ * *** **   ****  ****** ****** *   *** ** * ** 

Falni_camR      ACCATAATGAAACTGAATCGTTTTCATCGGTCTGGGGTGAATGACGCGACGATTTCCGGC
pSA3_camR       TCCATGAGCAAACTGAAACGTTTTCATCGCTCTGGAGTGAATACCACGACGATTTCCGGC
                 **** *  ******** *********** ***** ******  * **************

Falni_camR      AGTTTCTACTTATATATTAGCAAGATGTGGCGTGTTAAGGGGAAAACAAAACCTATTTCC
pSA3_camR       AGTTTCTACACATATATTCGCAAGATGTGGCGTGTTACGGTGAAAACCTGGCCTATTTCC
                *********  ******* ****************** ** ******    *********

Falni_camR      CTAAAGGGTTTATTGAGAATATGTTTTTCGTATCAGCCAATCCATGGGTGAGTTTCACCA
pSA3_camR       CTAAAGGGTTTATTGAGAATATGTTTTTCGTCTCAGCCAATCCCTGGGTGAGTTTCACCA
                ******************************* *********** ****************

Falni_camR      GTTTTGATTTAAACGTGGCCAATATGGACAACTTCTTCGCCCCCGTTTTCACCATGGGCA
pSA3_camR       GTTTTGATTTAAACGTGGCCAATATGGACAACTTCTTCGCCCCCGTTTTCACCATGGGCA
                ************************************************************

Falni_camR      AATATTATACGCAAGGCGACAAGGTGCTGATGCCGCTGGCGATTCAGGTTCATCATGCCG
pSA3_camR       AATATTATACGCAAGGCGACAAGGTGCTGATGCCGCTGGCGATTCAGGTTCATCATGCCG
                ************************************************************

Falni_camR      TTTGTGATGGCTTCCAAGTCGGCAGAATGCTTAATGAATTACAACAGTAATGCGATGAGT
pSA3_camR       TTTGTGATGGCTTCCATGTCGGCAGAATGCTTAATGAATTACAACAGTACTGCGATGAGT
                **************** ******************************** **********

Falni_camR      GGCAGGGCGGGGCCTAA
pSA3_camR       GGCAGGGCGGGGCGTAA
                ************* ***
```
